# Congenital factor XI deficiency caused by a novel F11 missense variant: a case report

**DOI:** 10.3325/cmj.2020.61.62

**Published:** 2020-02

**Authors:** Kristina Gotovac Jerčić, Antonela Blažeković, Mirea Hančević, Ervina Bilić, Fran Borovečki

**Affiliations:** 1 Department of Neurology, University Hospital Center Zagreb, Zagreb, Croatia; 2 Department of Anatomy, University of Zagreb School of Medicine, Zagreb, Croatia; 3 Center for Translational and Clinical Research, Department for Functional Genomics, University of Zagreb School of Medicine, Zagreb, Croatia

## Abstract

Hereditary factor XI (FXI) deficiency is a mild bleeding disorder, rare in the general population but relatively common among Ashkenazi Jews. The human *F11* gene comprises 15 exons, spanning over 23 kb of the long arm of chromosome 4 (4q35). Homozygotes or compound heterozygotes typically show severe FXI deficiency, whereas heterozygotes show partial or mild deficiency. However, the genotype-phenotype relationship is difficult to establish, even among individuals within the same family. We report on a female patient with a heterozygous variant in *F11* and FXI deficiency (49 IU/dL), who suffers from menorrhagia since menarche and easy bruising. She experienced excessive bleeding during thyroidectomy and after a cesarean section. Her younger sister, who carries the same heterozygous variant in *F11* and has mild FXI deficiency (47 IU/dL), has menorrhagia without other bleeding difficulties although she has undergone several surgeries. Their father, who carries the same missense variant, has not experienced any bleeding difficulties (but he has not undergone any surgeries either). The family study revealed that the A428C mutation was inherited from the father. This variant has not previously been described in the literature and is the first *F11* variant described in the Croatian population. Our study showed that even when family members have the same germline F11 variant, they still may experience phenotypic variability, making disease prognosis more complex.

Hereditary factor XI (FXI) deficiency is a rare condition in the general population (with estimated prevalence 1 per million) but relatively common in Ashkenazi Jews (1). The human *F11* gene consists of 15 exons and spans around 23 kb (2). Homozygotes or compound heterozygous have severe FXI deficiency (<15 IU/dL), while heterozygotes have partial or mild deficiency (20-70 IU/dL) (3). To date, 220 pathogenic variants in the *F11* gene have been reported, of which 57% are missense variants (4). However, it is difficult to determine the genotype-phenotype relationship, even among individuals from the same family, who despite having the same *F11* variant have different bleeding phenotypes (5). Here, we present a case of a novel heterozygous missense variant in *F11* gene in a Croatian family.

## CASE REPORT

One of the family members, a 27-year-old woman, first presented to our neurology clinic with upper limb weakness, difficulties releasing her hand-grip, and occasional leg cramps. She experienced excessive bleeding during thyroidectomy at the age of 26. Following a prolonged bleeding after a cesarean section at the age of 28, she was referred to a hematologist, who confirmed decreased FXI activity (49 IU/dL; reference range 67-127 IU/dL). She and two of her three sisters suffer from menorrhagia since menarche. Neurological examination revealed positive myotonic phenomenon in her hands, with no other neurological signs. Electromyography showed myotonic discharges in the hand muscles. Brain magnetic resonance imaging was normal, as were laboratory investigations, creatine kinase, protein electrophoresis, and acid alpha-glucosidase activity. Muscle biopsy was inconclusive, but she had bronchial spasm after inhalation anesthetic administration. Molecular genetic analysis excluded myotonic dystrophy type 1 and 2.

**Figure 1:**
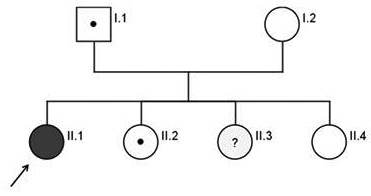
Family pedigree. Square indicates male members, circle indicates female members. Open symbols indicate unaffected individuals, dot in the middle indicates carriers, filled symbols indicate affected individuals, the arrow indicates the proband, the question mark represents the family member who was not tested.

**Figure 2:**
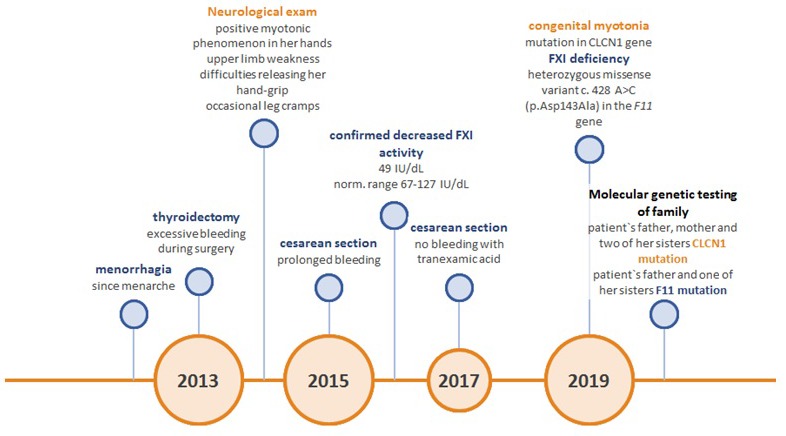
Medical history timeline.

Clinical exome sequencing identified a mutation in *CLCN1* gene, and the diagnosis of dominant congenital myotonia was established. Molecular genetic testing of her family revealed that her father, mother, and two of her sisters had *CLCN1* gene mutation. Her family history is positive for cramps, myotonic phenomenon, and muscle weakness in her father and her three sisters. Interestingly, a second mutation was found in the gene for FXI. The father had the FXI mutation and one sister had a decreased FXI activity and the FXI mutation (Figure 1, Figure 2).

**Figure 3:**
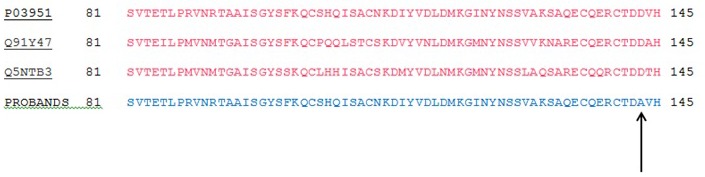
The location of the missense variant detected in the father and two daughters. The mutation site was referred against *F11* or *F11* homologue gene from *Homo sapiens* (P03951), *Mus musculus* (Q91y47), and *Bos Taurus* (Q5NTB3). The position of the missense mutation is marked by an arrow.

We performed clinical exome sequencing of the five probands (father, mother, and three sisters; one sister was not tested). An average of 30 million reads was generated per sample. Publicly available databases (dbSNP, 1000 Genomes Project, Database of Genomic Variants) were used to check for commonly known variants and exclude them from further analysis. A heterozygous missense variant c. 428 A>C (p.Asp143Ala) in the *F11* gene was selected as a potential disease-causing variant for the father and two daughters. *In silico* programs (MutationTaster, SIFT and Polyphen) confirmed that this variant was deleterious. The alignment of the three protein sequences showed that the novel F11 missense variant was located in an evolutionarily conserved region (Figure 3).

## DISCUSSION

We identified a novel missense *F11* variant in a Croatian family with FXI deficiency (father and two daughters). The family study revealed that the A428C mutation was inherited from the father. The novel variant c.428A>C leads to a sequence change that results in amino acid substitution (p.Asp143Ala) in the *F11* gene. This variant is not present in publicly available population databases. Missense variants in *F11* gene are known to be pathogenic and comprise the majority of identified genetic variants (6). *In silico* programs indicated that this variant was damaging, while sequence alignment programs showed that the residue p.Asp143 was phylogenetically conserved.

Heavy chain and the light chain of human FXI protein contain four apple (Ap) domains and a catalytic domain. The *F11* variant detected in this study is located in the Ap2 domain, which plays a major role in forming a complex with high molecular fibrinogen (7). In FXI-deficient individuals, pathogenic variants are rarest in the Ap2 domain when compared with the other three apple domains (4).

Our proband suffers from menorrhagia since menarche and easy bruising, while bleeding problems started during thyroidectomy and reappeared two years later after a cesarean section. Further hematological investigations confirmed FXI deficiency (49 IU/dL). Her younger sister, who also carries the same heterozygous variant in *F11* and has mild FXI deficiency (47 IU/dL), has menorrhagia without other bleeding difficulties. She underwent appendix removal, muscle biopsy, and scoliosis surgery without any bleeding incidents. The father, who carries the same missense variant, has not experienced any bleeding difficulties (but he has not undergone any surgeries either). Our patients showed intrafamilial phenotype variability, which is consistent with previous findings (8). Bleeding and FXI levels show a weak correlation, and genotype-phenotype relationships have been difficult to define (9,10). This is also the case in our patients, since the younger sister with the same variant and slightly lower FXI level than our proband has only menorrhagia but has had no bleedings during or after surgeries.

Postpartum hemorrhage occurs more frequently if the patient has a history of bleeding, so pregnant women with bleeding phenotype are administered FXI concentrate or fresh frozen plasma at delivery. Our proband experienced prolonged bleeding after the first cesarean section, so before the second cesarean section she received antifibrinolytic tranexamic acid and fresh frozen plasma during surgery. The second cesarean section was without prolonged bleeding, but she experienced postpartum wound inflammation followed by fever.

In conclusion, we identified a novel missense variant c.428A>C in the *F11* gene in a father and two daughters. This is the first *F11* variant described in the Croatian population. According to available public databases and current literature, the variant has also not been reported in other populations. Our study showed that even when the same germline *F11* variant is identified in a family, the patients still experience phenotypic variability, making the disease prognosis more complex.
